# Impact of surgical delay on bowel viability in internal hernia-related small bowel obstruction: a retrospective cohort study of clinical, imaging, and biochemical predictors of strangulation

**DOI:** 10.1186/s12876-026-04835-0

**Published:** 2026-04-21

**Authors:** Judong Zhang, Yifang Hsieh, Longchun Dong, Jing Xu

**Affiliations:** 1 Department of General Surgery, Tianjin Union Medical Center, The First Affiliated Hospital of Nankai University, Tianjin, 300121 China; 2 Tianjin Key Laboratory of General Surgery in construction, Tianjin Union Medical Center, Tianjin, 300121 China; 3 School of Medicine, Nankai University, Tianjin, 300071 China; 4 Department of Radiology, Tianjin Union Medical Center, The First Affiliated Hospital of Nankai University, Tianjin, 300121 China

**Keywords:** Internal hernia, Small bowel obstruction, Strangulation, Base excess, Computed tomography

## Abstract

**Background:**

Internal hernia–related small bowel obstruction (SBO) is a time-critical condition that may rapidly progress to strangulation and bowel necrosis. Early surgical decision-making is challenging because classical clinical and radiological signs of ischemia are often absent during the reversible phase. This study aimed to identify clinical, imaging, and biochemical factors associated with bowel strangulation and delayed surgical intervention in patients with internal hernia–related SBO.

**Methods:**

This retrospective cohort study included patients who underwent surgery for internal hernia–related SBO between January 2019 and December 2025. Admission laboratory parameters and preoperative non-enhanced abdominal computed tomography (CT) findings were analyzed. Multivariable logistic regression was used to identify predictors of bowel strangulation and factors associated with delayed surgical intervention. Clinical outcomes were compared between delayed and non-delayed surgery groups.

**Results:**

A total of 119 patients were included, of whom 82 (68.9%) had strangulated internal hernia. Lactate (OR = 3.975) and D-dimer (OR = 3.412) were independent predictors of bowel strangulation. In patients with strangulated internal hernia, the absence of peritonitis (OR = 0.021), absence of the whirlpool sign on CT (OR = 0.147), and higher base excess (OR = 1.274) were independently associated with delayed surgical intervention. Patients in the delayed surgery group had significantly higher rates of bowel resection (85.7% vs. 53.2%), bowel necrosis, and longer hospital stays compared with those undergoing timely surgery.

**Conclusion:**

Surgical delay in internal hernia–related SBO is frequently driven by deceptively mild clinical and radiological findings during early ischemia. While lactate and D-dimer indicate established strangulation, base excess—together with non-enhanced CT features—may provide earlier warning of mesenteric compromise. An integrated interpretation of laboratory and imaging findings may facilitate earlier surgical intervention, reduce bowel resection, and improve clinical outcomes.

**Supplementary Information:**

The online version contains supplementary material available at https://doi.org/10.1186/s12876-026-04835-0.

## Background

Small bowel obstruction (SBO) is a common and clinically important cause of acute abdomen [1], and despite the wide spectrum of etiologies, internal hernias account for only a small proportion overall [2]. Within adhesive etiologies, however, internal herniation caused by a single adhesive band represents a distinct pattern. In some surgically confirmed cases of internal hernia-related small bowel obstruction, an isolated adhesive band may create an abnormal intra-abdominal aperture that traps the bowel and produces a hernia-like closed-loop configuration with a high risk of strangulation. Compared with diffuse adhesion complexes, this fixed, two-point tethering of the bowel predisposes to rapid compromise of mesenteric perfusion and progression to strangulated SBO (SSBO) [3, 4]. The core clinical difficulty lies in the early ischemic phase, where mesenteric perfusion is compromised but transmural necrosis has not yet developed, and clinical findings remain indistinguishable from simple obstruction. Once signs of peritonitis appear, irreversible necrosis has often already developed, leaving very limited opportunities for bowel salvage [5, 6].

Computed tomography (CT) is considered the diagnostic cornerstone for SBO [7], yet non-contrast CT is still commonly performed in emergency practice due to renal impairment, contrast allergy, hemodynamic instability, or resource limitations. However, its specificity for detecting subtle ischemic changes is markedly lower than that of contrast-enhanced CT [8]. As a result, operative timing in real-world settings continues to rely heavily on the progression of abdominal pain and late clinical deterioration, which may delay necessary intervention and increase the likelihood of bowel resection. These limitations highlight a critical gap in current diagnostic strategies: the need for a practical method capable of identifying ischemia during the reversible window, before necrosis and peritonitis occur.

This study aimed to evaluate clinical, imaging, and biochemical factors associated with bowel strangulation and delayed surgical intervention in patients with internal hernia-related small bowel obstruction.

## Methods

### Study population

This retrospective cohort study was designed and reported in accordance with the STROBE (Strengthening the Reporting of Observational Studies in Epidemiology) guidelines and conducted in accordance with the Declaration of Helsinki. We retrospectively reviewed the clinical data of 184 patients who underwent surgical treatment for small bowel obstruction caused by intra-abdominal hernia at Tianjin Union Medical Center between January 2019 and December 2025. The inclusion criteria were as follows: (1) confirmed diagnosis of small bowel obstruction requiring hospitalization; (2) receipt of surgical treatment; and (3) definitive diagnosis confirmed by operative findings and intraoperative records. The exclusion criteria were: (1) conditions affecting coagulation function, including coagulation disorders, advanced malignancy, or menstruation; (2) conditions affecting serum albumin (ALB) levels, such as liver cirrhosis, malnutrition, or advanced malignancy; and (3) incomplete laboratory or radiology data. A total of 184 patients were initially identified and classified into non-strangulated and strangulated groups according to disease severity. After applying the exclusion criteria, 119 patients were ultimately included in the final analysis. Demographic characteristics (age, sex, height, and weight), time from symptom onset, and relevant clinical and laboratory data were collected for all included patients. Patients with incomplete key laboratory or radiological data were excluded during cohort assembly; therefore, analyses were performed on complete cases, and no additional imputation was undertaken. In addition, because only patients who underwent surgery and had intraoperatively confirmed internal hernia were included, non-operatively managed cases and potentially missed cases were not captured. Therefore, the findings of this study are primarily generalizable to surgically treated internal hernia-related small bowel obstruction, and the observed prevalence of strangulation may be overestimated relative to an unselected clinical population.

### Variables and data collection

Laboratory data obtained from the first blood tests after admission were collected, including complete blood count parameters (white blood cell count(WBC), neutrophils, lymphocytes, neutrophil percentage, platelet), biochemical indices (Albumin, Creatine Kinase(CK), Creatine kinase-MB(CKMB), lactate dehydrongenase (LDH), blood urea nitrogen(BUN)), coagulation-related markers (D-dimer, fibrinogen), and arterial blood gas parameters ( base excess [BE], and lactate [LAC]). Derived inflammatory indices, including the neutrophil-to-lymphocyte ratio (NLR) and platelet-to-lymphocyte ratio (PLR), were calculated accordingly. To improve measurement comparability across groups, laboratory variables were collected from the first available blood tests after admission.

Surgical delay was operationally defined in this study as an interval exceeding six hours between emergency computed tomography (CT) acquisition and surgical intervention. This cutoff was based on institutional emergency surgical workflow. In patients determined to require immediate emergency surgery after CT evaluation, the entire process—including surgical decision-making, preoperative preparation, and hospital admission—was typically completed within six hours at our institution. This interval therefore represented the usual clinical workflow for timely emergency surgery. In contrast, patients not undergoing surgery within this period generally experienced additional observation or delay in operative decision-making. Therefore, the six-hour threshold was chosen to distinguish timely intervention from delayed management, while also reflecting previously reported early operative windows in strangulated small bowel obstruction.

### CT imaging assessment

**Fig. 1:**
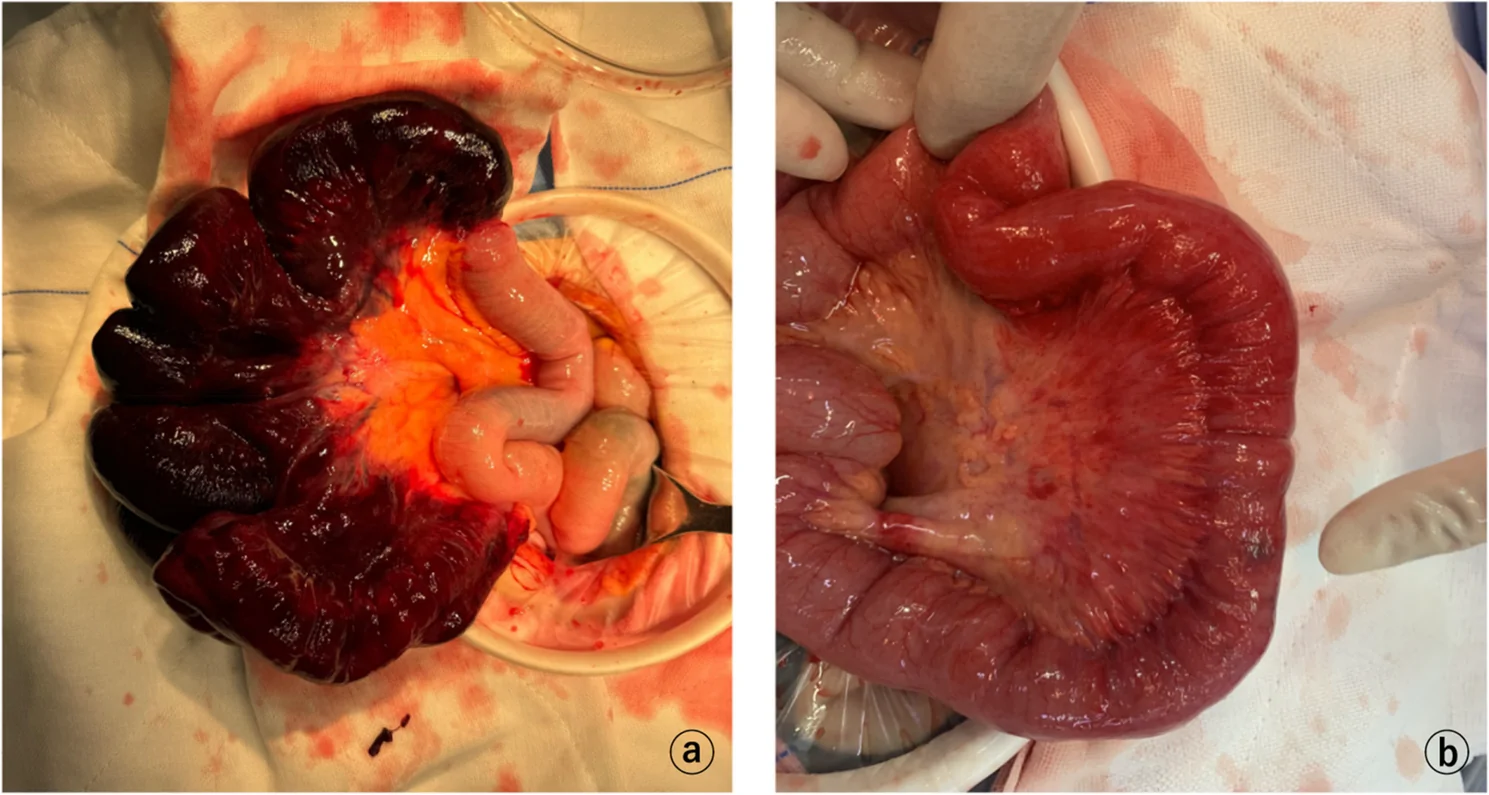
Intraoperative images. **A** delayed surgical intervention resulting in bowel necrosis. **B** timely surgical release of strangulation with restoration of bowel color and viability

**Fig. 2:**
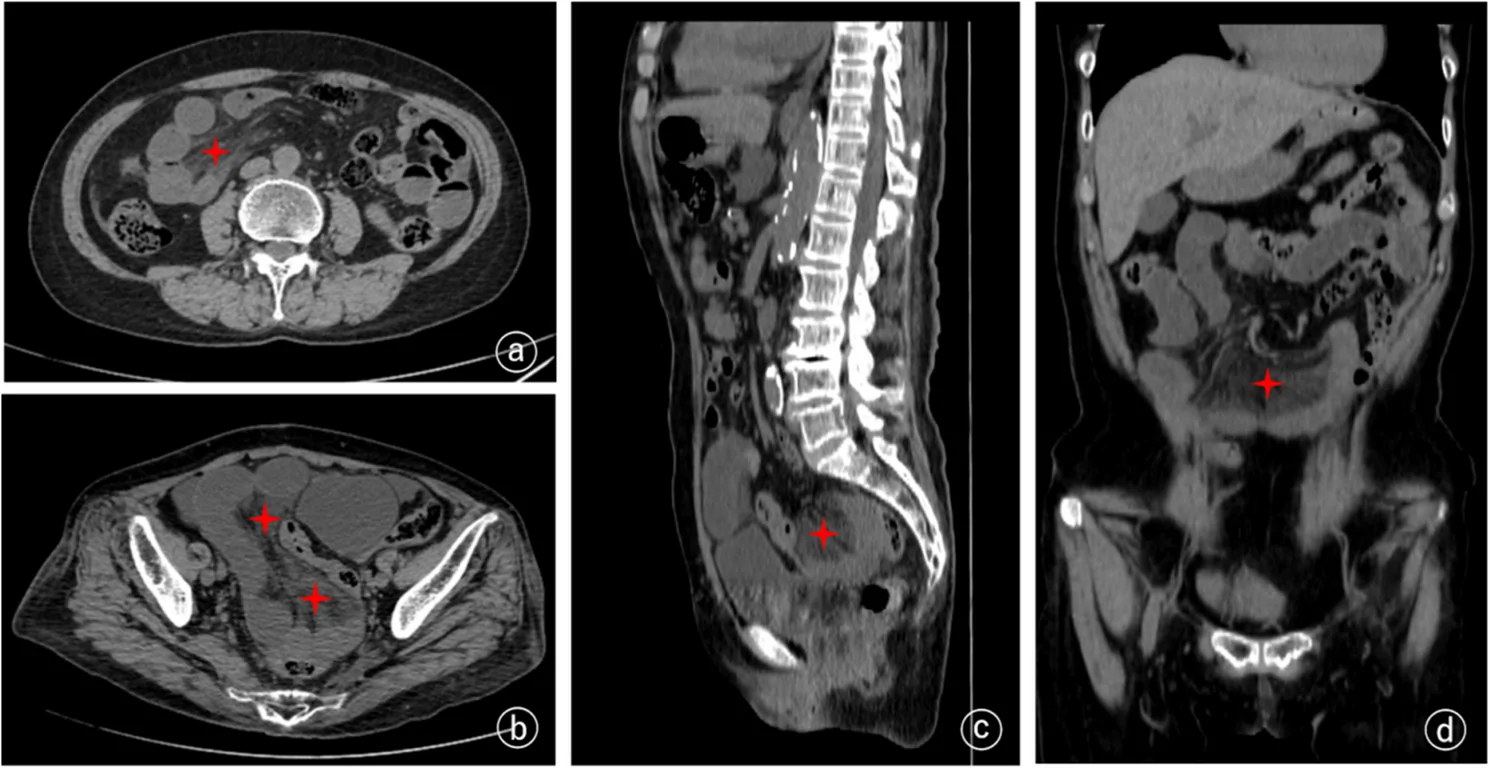
CT manifestations of mesentric edema red four-pointed stars: mesenteric edema; **A**, **B** Axial views; **C** sagittal view; **D** coronal view

**Fig. 3:**
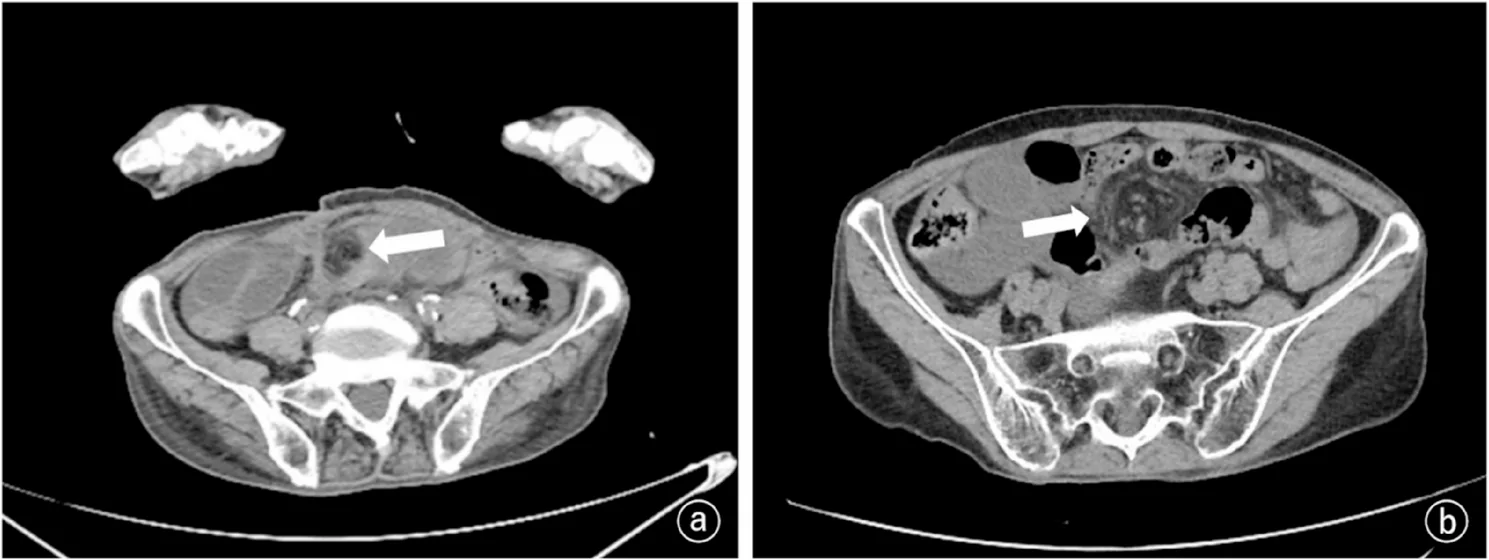
Whirlpool sign. Both **a** and **b** show the whirlpool sign (white arrows), indicating mesenteric torsion

**Fig. 4:**
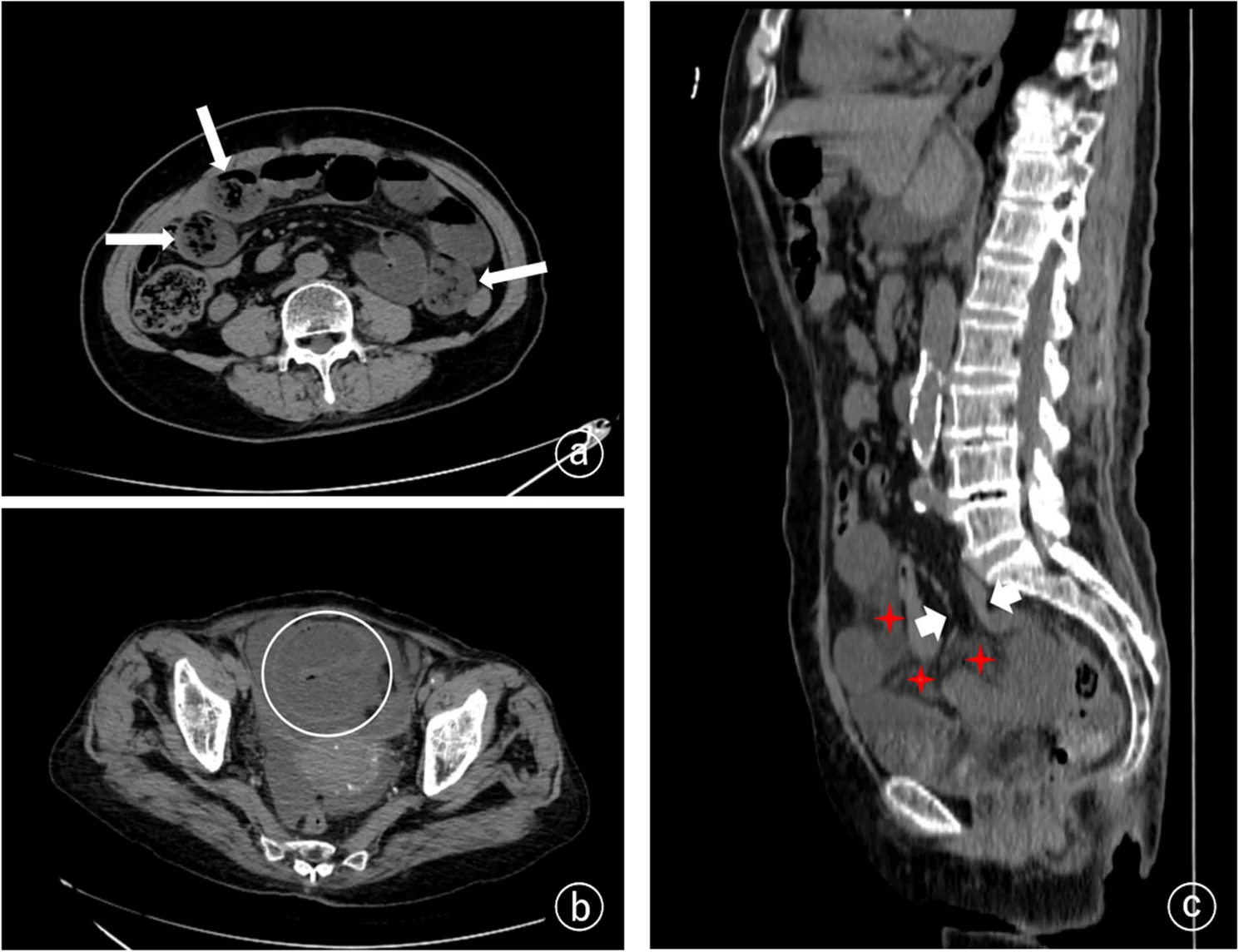
Characteristic CT signs of strangulated small bowel obstruction **a** small bowel feces sign (thick white arrow) ; **b** coffee bean sign (white circle) ; **c** mesenteric edema (red four-pointed star) and hernia orifice (thick white arrow)

All CT examinations in this cohort were non-contrast scans performed as part of routine emergency evaluation. CT images were interpreted by attending radiologists in routine clinical practice, and the surgeons reviewed the images as part of preoperative assessment. CT findings analyzed in this study were recorded according to the initial clinical reports. Imaging assessment focused on the presence of small bowel obstruction and radiological signs suggestive of strangulation or ischemia. The evaluated CT features included bowel wall thickening, altered bowel wall attenuation (increased or decreased), small bowel feces sign, mesenteric edema or stranding, ascites, mesenteric fluid accumulation, pneumatosis intestinalis, portal venous or mesenteric venous gas, free intraperitoneal air, and intraluminal hemorrhage. Morphological signs of closed-loop obstruction were also assessed, including the coffee bean sign, U-shaped or C-shaped bowel loops, and beak sign. In addition, specific mesenteric vascular findings such as the whirlpool sign and mesenteric crowding signs (including the comb sign and cable sign) were recorded. CT findings were documented based on initial clinical reports and correlated with intraoperative findings for diagnostic confirmation. CT interpretation was based on routine clinical reports to reflect real-world diagnostic practice. The absence or presence of key radiological signs was subsequently analyzed in relation to surgical timing and clinical outcomes. To improve measurement comparability across groups, CT findings were extracted from the initial preoperative non-contrast CT reports, and bowel viability was classified according to operative findings using the same institutional criteria throughout the study period. Representative operative and CT images are shown in Figs. 1, 2, 3 and 4.

### Bias

Several measures were taken to address potential bias. Consecutive eligible surgical patients during the study period were included to reduce selection bias. Laboratory, imaging, and operative variables were extracted using predefined definitions to reduce information bias. Multivariable logistic regression was performed to account for potential confounding; however, residual confounding, including confounding by indication related to real-world surgical decision-making, cannot be fully excluded.

### Statistical analysis

All statistical analyses were performed using SPSS software (version 27.0). Continuous variables were assessed for normality prior to analysis. Data with a normal distribution are presented as mean ± standard deviation (SD) and were compared using the independent-samples t test. Non-normally distributed data are presented as median with interquartile range [M (P25, P75)] and were compared using the Mann–Whitney U test. Categorical variables are presented as frequencies and percentages and were analyzed using the chi-square test, with continuity correction applied when appropriate. Variables identified as statistically significant in univariable logistic regression were further examined for multicollinearity prior to inclusion in the multivariable model. Multicollinearity was assessed using tolerance statistics and variance inflation factors (VIF), with a VIF ≤ 5 indicating no substantial multicollinearity. Variables meeting this criterion were subsequently incorporated into the multivariable regression analysis. To reduce the risk of overfitting, the number of covariates was restricted with consideration of events-per-variable (EPV) principles and clinical relevance. No formal sample size calculation was performed because of the retrospective design. The study size was determined by the number of consecutive eligible patients with surgically confirmed internal hernia treated at our institution during the study period.

## Results

### Patient characteristics

**Fig. 5:**
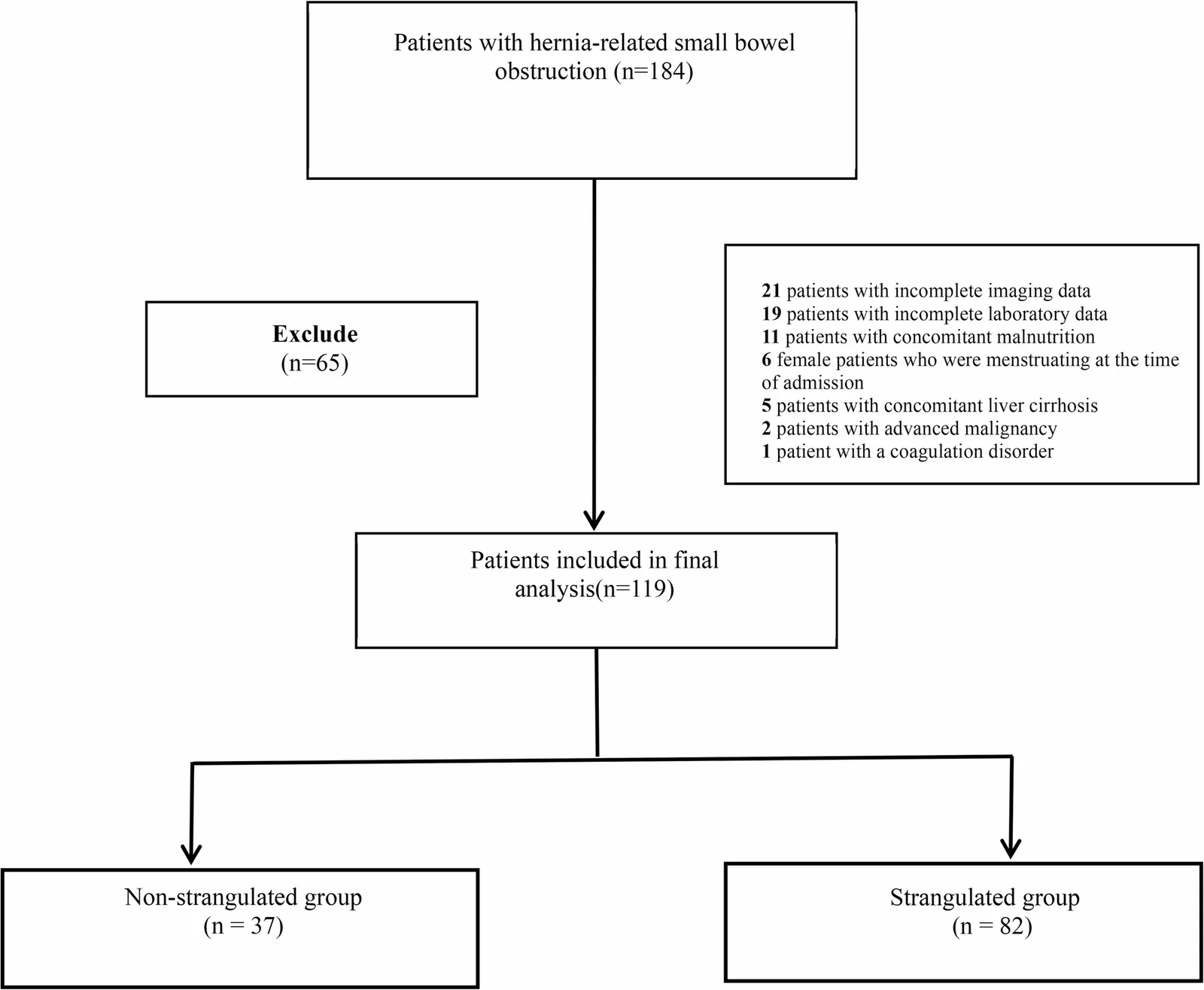
Participant flow chart of the study flow diagram showing the number of patients screened, excluded, and included in the final analysis, as well as classification into non-strangulated and strangulated groups

During the study period, 184 surgically treated patients with small bowel obstruction caused by intra-abdominal hernia were retrospectively screened. After exclusion of patients who did not meet the eligibility criteria or had incomplete data, 119 patients with confirmed internal hernia-related small bowel obstruction were included in the final analysis. (Fig. 5) The demographic and clinical characteristics are summarized in Table 1. The cohort consisted of 54 males (45.4%) and 65 females (54.6%), with a median age of 68 years (interquartile range [IQR]: 57.0–75.0 years). A significant majority of patients (68.9%) had a history of abdominal surgery. Regarding clinical presentation, abdominal pain was present in all 119 patients (100%), while abdominal distention and nausea/vomiting occurred in 79% and 79.8% of cases, respectively. Among the entire cohort, 82 patients (68.9%) were diagnosed with strangulated IH-related small bowel obstruction, of whom 53 (44.5%) presented with bowel necrosis. Surgical delay occurred in 37 patients (31.1%). The median postoperative hospital stay was 12 days (IQR: 9–17 days), with an overall mortality rate of 5%.

**Table 1:** Patient characteristics. Baseline demographic, clinical, laboratory, and outcome characteristics of the study population

Variables	Patients (*n* = 119)
Sex, n(%)	
Male	54(45.4%)
Female	65(54.6%)
Age(years), M(Q₁,Q₃)	68(57.0,75.0)
Height(cm), M(Q₁,Q₃)	165(160.0,170.0)
Weight(kg), M(Q₁,Q₃)	58(51.0,65.0)
BMI(Kg/m²), M(Q₁,Q₃)	21.5(19.5,23.2)
Abdominal Surgery History, n(%)	
Yes	82(68.9%)
No	37(31.1%)
Number of Abdominal Surgeries, M(Q₁,Q₃)	1(0,1)
Abdominal Pain, n(%)	
Yes	119(100%)
No	0(0%)
Abdominal Distension, n(%)	
Yes	94(79%)
No	25(21%)
Nausea or vomiting, n(%)	
Yes	95(79.8%)
No	24(20.2%)
Cessation of Flatus and Defecation, n(%)	
Yes	82(68.9%)
No	37(31.1%)
Fever, n(%)	
Yes	6(5%)
No	113(95%)
Peritonitis, n(%)	
Yes	30(25.2%)
No	89(74.8%)
Strangulation, n(%)	
Yes	82(68.9%)
No	37(31.1%)
Bowel Perforation, n(%)	
Yes	9(7.6%)
No	110(92.4%)
Bowel Stenosis, n(%)	
Yes	15(12.6%)
No	104(87.4%)
Time from onset to admission (hours), M(Q₁,Q₃)	24(12, 96)
Time from onset to surgery(hours), M(Q₁,Q₃)	72.2(26, 240)
Surgical Procedure, n(%)	
Adhesiolysis	51(42.9%)
Partial Small Bowel Resection	68(57.1%)
Bowel Resection, n(%)	
Yes	51(42.9%)
No	68(57.1%)
Total Hospital Days(days), M(Q₁,Q₃)	17(10, 22)
Postoperative hospital Stay(days), M(Q₁,Q₃)	12(9, 17)
ICU admission, n(%)	
Yes	18(15.1%)
No	101(84.9%)
Mortality, n(%)	
Yes	6(5%)
No	113(95%)

Baseline demographic, clinical, laboratory, and outcome characteristics of the study population

### Risk factors for strangulated internal hernia

Univariate and multivariate logistic regression analyses were performed to identify predictors of bowel strangulation (Table 2). Univariate analysis revealed that time from onset to admission, small bowel feces sign, mesenteric fluid, closed-loop obstruction, and several laboratory parameters—including WBC count, neutrophil count, NLR, PLR, LAC, BE, LDH, BUN, and D-dimer—were significantly associated with strangulation (all *P* < 0.05). Multicollinearity diagnostics were subsequently performed for variables identified as statistically significant in the univariable analysis (Supplementary Table 1). The results demonstrated that white blood cell count (VIF = 94.391), neutrophil count (VIF = 116.639), and the neutrophil-to-lymphocyte ratio (NLR; VIF = 6.765) had variance inflation factors exceeding 5, indicating substantial multicollinearity; therefore, these variables were excluded from further analysis. The remaining variables showed tolerance values ranging from 0.424 to 0.800 and VIF values ranging from 1.250 to 3.440, all meeting the predefined criterion of VIF ≤ 5, suggesting no significant multicollinearity. These variables were subsequently entered into the multivariable logistic regression model.

**Table 2:** Risk factors for strangulated internal hernia-related SBO. Univariate and multivariable analyses of factors associated with bowel strangulation in patients with internal hernia-related small bowel obstruction

Variables	Univariate analysis(*n* = 119)			Multivariate analysis(*n* = 119)	
	Non-strangulated (*n* = 37)	Strangulated (*n* = 82)	*p*-value	OR (95%CI)	*p*-value
Sex			0.933		
Male	17(45.9%)	37(54.9%)			
Female	20(54.1%)	45(45.1%)			
Age	65.75 ± 11.44	67.43 ± 9.76	0.151		
Height(cm)	170 ± 8.49	164.86 ± 9.76	0.463		
Weight(kg)	62.00 ± 10.30	59.86 ± 12.37	0.189		
BMI (kg/m^2^)	21.35 ± 2.16	21.91 ± 3.19	0.168		
Abdominal Surgery History			0.832		
Yes	25(67.6%)	57(69.5%)			
No	12(32.4)	25(30.5%)			
Number of Abdominal Surgeries	1(0, 1)	1(0, 1)	0.482		
Time from onset to admission(h)	66.00 ± 40.99	42.71 ± 26.71	< 0.001	0.984(0.973–0.995)	0.004
Abdominal Pain			-		
Yes	37(100%)	82(100%)			
No	0(0%)	0(0%)			
Abdominal Distension			0.075		
Yes	33(89.2%)	61(74.4%)			
No	4(10.8%)	21(25.6%)			
Nausea and/or Vomiting			0.449		
Yes	28(75.7%)	67(81.7%)			
No	9(24.3%)	15(18.3%)			
Cessation of Flatus and Defecation			0.058		
Yes	30(81.1%)	52(63.4%)			
No	7(18.9%)	30(36.6%)			
Fever			0.999		
Yes	0(0%)	6(7.3%)			
No	37(100%)	76(92.7%)			
Peritonitis			0.998		
Yes	0(0%)	30(36.6%)			
No	37(100%)	52(63.4%)			
Small bowel Feces sign			< 0.001	3.471(0.742–15.681)	0.115
Yes	5(13.5%)	47(57.3%)			
No	32(86.5%)	35(42.7%)			
Bowel wall Thickening			0.395		
Yes	11(29.7%)	51(62.2%)			
No	26(70.3%)	31(37.8%)			
Increased Bowel Wall Attenuation			0.999		
Yes	0(0%)	10(13.9%)			
No	37(100%)	72(86.1%)			
Decreased Bowel Wall Attenuation			0.759		
Yes	10(27.0%)	20(24.4%)			
No	27(73.0%)	62(75.6%)			
Mesenteric Stranding			0.001	0.535(0.047–6.028)	0.613
Yes	26(70.3%)	77(93.9%)			
No	11(29.7%)	5(6.1%)			
Peritoneal Fluid			0.058		
Yes	26(70.3%)	70(85.4%)			
No	11(29.7%)	12(14.6%)			
Intraluminal Hemorrhage			0.999		
Yes	0(0%)	4(4.9%)			
No	37(100%)	78(95.1%)			
Pneumatosis Intestinalis			0.999		
Yes	0(0%)	4(4.9%)			
No	37(100%)	78(95.1%)			
Portal or mesenteric Venous Gas			-		
Yes	0(0%)	0(0%)			
No	37(100%)	82(100%)			
Free Intraperitoneal Air			-		
Yes	0(0%)	5(6.1%)			
No	37(100%)	77(93.9%)			
Coffee Bean Sign; U Loop; C Loop			0.005	0.830(0.220–3.127)	0.783
Yes	15(40.5%)	56(68.3%)			
No	22(59.5%)	26(31.7%)			
Target Sign			0.184		
Yes	5(13.5%)	62(75.6%)			
No	32(86.5%)	20(24.4%)			
Whirlpool Sign			0.169		
Yes	4(10.8%)	13(15.8%)			
No	33(89.2%)	69(84.2%)			
Mesenteric Crowding: Rope Sign; Comb Sign			0.755		
Yes	12(32.4%)	29(35.4%)			
No	25(67.6%)	53(64.6%)			
Bird’s Peak Sign			0.742		
Yes	32(86.5%)	69(84.2%)			
No	5(13.5%)	13(15.8%)			
White Blood Cell Count	7.88 ± 4.08	10.48(8.12, 12.99)	< 0.001		
Neutrophil(N)	5.70 ± 3.87	8.70(7.09, 11.62)	< 0.001		
Lymphocyte(L)	1.58 ± 0.50	0.83 ± 0.40	0.144		
N%	67.05 ± 15.11	87.90(83.58, 90.05)	< 0.001	1.049(0.998–1.102)	0.06
NLR (N/L)	4.05 ± 3.06	13.43(9.02, 17.53)	< 0.001		
PLR (Platelet to lymphocyte ratio)	139.73 ± 49.48	247.99(183.88, 389.58)	0.006	1.003(0.998–1.007)	0.285
Platelet	206.25 ± 58.81	213.07 ± 59.16	0.786		
Lactate	1.25 ± 0.51	2.41 ± 0.97	< 0.001	3.975(1.294–12.211)	0.027
Base Excess (BE)	-0.3 ± 0.70	-3.73 ± 3.83	< 0.001	0.772(0.587–1.016)	0.065
Lactate Dehydrogenase	207.5 ± 25.3	240(210.50, 256.75)	0.042		
Creatine Kinase (CK)	74.5 ± 30.09	97.21 ± 57.68	0.100		
CKMB	7.88 ± 2.35	6.71 ± 5.06	0.306		
Potassium	4.02 ± 0.55	4.13 ± 0.66	0.338		
Sodium	139.55 ± 2.67	137.75 ± 4.89	0.379		
Blood Urea Nitrogen	4.37 ± 1.79	9.58 ± 5.61	0.002	1.169(0.940–1.516)	0.146
Albumin	37.35 ± 2.74	38.91 ± 7.13	0.102		
D-dimer	0.65 ± 0.49	2.05 ± 1.92	0.003	3.412(1.070-10.879)	0.008
Fibrnogen	3.05 ± 0.73	4.53 ± 2.23	0.537		

Univariate and multivariable analyses of factors associated with bowel strangulation in patients with internal hernia-related small bowel obstruction

Multivariable logistic regression analysis revealed that duration of symptoms before admission (OR = 0.984, 95% CI: 0.973–0.995, *P* = 0.004), lactate level (OR = 3.975, 95% CI: 1.294–12.211, *P* = 0.027), and D-dimer level (OR = 3.412, 95% CI: 1.070–10.879, *P* = 0.038) were independently associated with intestinal strangulation.

## Factors associated with surgical delay in strangulated IH-related small bowel obstruction

In the subset of 82 patients with strangulated IH- related small bowel obstruction, factors associated with surgical delay were analyzed (Table 3). Univariate analysis showed significant differences between the delayed and non-delayed groups regarding peritonitis (*P* < 0.001), the whirlpool sign (*P* = 0.045), base excess (*P* = 0.027), and fibrinogen (*P* = 0.014). Multivariate analysis identified the absence of peritonitis (OR = 0.021, 95% CI: 0.003–0.130, *P* < 0.001) and the absence of the whirlpool sign (OR = 0.147, 95% CI: 0.023–0.947, *P* = 0.044) as independent risk factors for surgical delay. Additionally, base excess remained a significant factor in the multivariate model (OR = 1.274, 95% CI: 1.040–1.562, *P* = 0.020).

**Table 3:** Factors associated with surgical delay in strangulated IH-related SBO. Univariate and multivariable analyses of factors associated with delayed surgical intervention among patients with strangulated internal hernia-related small bowel obstruction

Variables	Univariate analysis(*n* = 82)			Multivariate analysis(*n* = 82)	
	Non-Delayed Surgery (*n* = 47)	Delayed surgery(*n* = 35)	*p*-value	OR (95%CI)	*p*-value
Sex			0.722		
Male	22(46.8%)	15(57.1%)			
Female	25(53.2%)	20(42.9%)			
Age	65.38 ± 15.23	70.5(62.5, 76)	0.326		
Height(cm)	165(159, 168)	166.09 ± 7.46	0.426		
Weight(kg)	57(50, 65)	57.75(50, 70)	0.422		
BMI (kg/m^2^)	21.19 ± 2.06	21.60 ± 2.84	0.585		
Abdominal Surgery History			0.419		
Yes	31(66.0%)	26(74.3%)	0.655		
No	16(34.0%)	9(25.7%)	0.187		
Number of Abdominal Surgeries	1(0, 1)	1(0, 1)	0.655		
Abdominal Pain			0.816		
Yes	34(72.3%)	27(22.9%)			
No	13(27.7%)	8(77.1%)			
Abdominal Distension			0.623		
Yes	34(72.3%)	27(22.9%)			
No	13(27.7%)	8(77.1%)			
Nausea and/or Vomiting			0.816		
Yes	38(80.9%)	29(82.9%)			
No	9(19.1%)	6(17.1%)			
Cessation of Flatus and Defecation			0.196		
Yes	27(57.4%)	25(67.6%)			
No	20(42.6%)	10(32.4%)			
Fever			0.212		
Yes	5(10.6%)	1(2.9%)			
No	42(89.4%)	34(97.1%)			
Peritonitis			< 0.001	0.021(0.003–0.130)	< 0.001
Yes	28(59.6%)	2(5.7%)			
No	19(40.4%)	33(94.3%)			
Small bowel Feces sign			0.069		
Yes	31(66.0%)	16(45.7%)			
NO	16(34.0%)	19(54.3%)			
Bowel wall Thickening			0.204		
Yes	15(40.5%)	16(45.7%)			
No	32(59.5%)	19(54.3%)			
Increased Bowel Wall Attenuation			0.619		
Yes	5(10.6%)	5(14.3%)			
No	42(89.4%)	30(85.7%)			
Decreased Bowel Wall Attenuation			0.204		
Yes	9(19.1%)	11(31.4%)			
No	38(80.9%)	24(68.6%)			
Mesenteric Stranding			0.313		
Yes	43(91.5%)	34(97.1%)			
No	4(8.5%)	1(2.9%)			
Peritoneal Fluid			0.939		
Yes	40(85.1%)	30(85.7%)			
No	7(14.9%)	5(14.3%)			
Intraluminal Hemorrhage			0.214		
Yes	1(2.1%)	3(8.6%)			
No	46(97.9%)	32(91.4%)			
Pneumatosis Intestinalis					
Yes	1(2.1%)	3(8.6%)	0.214		
No	46(97.9%)	32(91.4%)			
Portal or mesenteric Venous Gas			-		
Yes	0(0%)	0(0%)			
No	47(100%)	35(100%)			
Free Intraperitoneal Air			0.428		
Yes	2(4.2%)	3(8.6%)			
No	45(95.8%)	32(91.4%)			
Coffee Bean Sign; U Loop; C Loop			0.363		
Yes	34(72.3%)	22(62.9%)			
No	13(27.7%)	13(37.1%)			
Target Sign			0.426		
Yes	34(72.3%)	28(80.0%)			
No	13(27.7%)	7(20.0%)			
Whirlpool Sign				0.147(0.023–0.947)	0.044
Yes	11(23.4%)	2(5.7%)	0.045		
No	36(76.6%)	33(94.3%)			
Mesenteric Crowding: Rope Sign; Comb Sign			0.450		
Yes	15(40.5%)	14(40.0%)			
No	32(59.5%)	21(60.0%)			
Bird’s Peak Sign			0.738		
Yes	39(83.0%)	30(85.7%)			
No	8(17.0%)	5(14.3%)			
White Blood Cell Count	12.13 ± 4.77	9.58(7.86, 12.78)	0.117		
Neutrophil(N)	10.80 ± 4.70	8.39(6.81, 11.25)	0.119		
Lymphocyte(L)	0.88(0.60,1.07)	0.69(0.41, 1.13)	0.169		
N%	90(82.5, 92.6)	87.75(84.35, 91.43)	0.496		
NLR (N/L)	14.78 ± 9.21	13.48(8.60, 18.66)	0.968		
PLR (Platelet to lymphocyte ratio)	263.33(201.24, 371.67)	280.15(216.08, 455.96)	0.230		
Platelet	223(191, 252)	204(178.00, 267.25)	0.954		
Lactate	2.30(1.59, 3.30)	2.10(1.30, 2.93)	0.449		
Base Excess(BE)	-3.5(-5.2, -2.7)	-2.47 ± 3.47	0.027	1.274(1.040–1.562)	0.020
Lactate Dehydrogenase	235(192, 300)	234.50(297.25,255.25)	0.273		
Creatine Kinase(CK)	64(49, 95)	81.26 ± 44.87	0.815		
CKMB	7.19(2.90,11.00)	6.10(3.20, 11.95)	0.338		
Potassium	4.09(3.78, 4.36)	3.96 ± 0.59	0.169		
Sodium	138.97 ± 3.34	138.90(136.83, 141.43)	0.383		
Blood Urea Nitrogen	8.02 ± 2.85	6.53(4.73, 9.81)	0.479		
Albumin	41.70(36.80, 45.60)	38.20 ± 7.14	0.082		
D-dimer	0.96(0.64, 2.50)	1.27(0.61,2.93)	0.193		
Fibrnogen	3.06(2.65,3.90)	3.76(2.82, 4.56)	0.014	1.689(0.940–3.037)	0.080

Univariate and multivariable analyses of factors associated with delayed surgical intervention among patients with strangulated internal hernia-related small bowel obstruction

### Prognostic comparison between delayed and non-delayed groups

The clinical outcomes of the surgical delay group (*n* = 35) were significantly poorer than those of the non-delayed group (*n* = 47) (Table 4). The delay group exhibited significantly higher rates of bowel resection (85.7% vs. 53.2%, *P* = 0.002), bowel necrosis (80.0% vs. 53.2%, *P* = 0.012), and bowel perforation (20.0% vs. 4.3%, *P* = 0.033). Furthermore, the total hospital stays (18 vs. 11 days, *P* < 0.001) and postoperative hospital stay (17 vs. 11 days, *P* = 0.003) were significantly longer in the delay group. Although the mortality rate and ICU admission rate were numerically higher in the delay group, these differences did not reach statistical significance (all *P* > 0.05).

**Table 4:** Prognostic comparison between delayed and non-delayed surgery groups. Comparison of clinical outcomes between delayed and non-delayed surgical intervention groups among patients with strangulated internal hernia-related small bowel obstruction

Variables	Non-delayed surgery (*n* = 47)	Delayed Surgery (*n* = 35)	*p*-value
Surgical Procedure			0.002
Adhesiolysis	22(46.8%)	5(14.3%)	
Partial Small Bowel Resection	25(53.2%)	30(85.7%)	
Ischemic Necrosis			0.012
Ischemia	22(46.8%)	7(20.0%)	
Necrosis	25(53.2%)	28(80.0%)	
Perforation			0.033
Yes	2(4.2%)	7(20.0%)	
No	45(95.8%)	28(80.0%)	
Stenosis			1.0
Yes	2(4.2%)	1(2.9%)	
NO	45(95.8%)	34(97.1%)	
Total Hospital Stay (days)	11(8, 17)	18(12, 28)	< 0.001
Postoperative Hospital Stay (days)	11(8, 14)	17(11, 22)	0.003
ICU admission			0.211
Yes	8(17.8%)	10(28.6%)	
none	39(82.2%)	25(71.4%)	
Mortality			1.0
Yes	3(6.4%)	2(5.7%)	
none	44(93.6%)	33(94.3%)	
Bowel Resection			0.002
Yes	25(53.2%)	30(85.7%)	
none	22(46.8%)	5(14.3%)	

Comparison of clinical outcomes between delayed and non-delayed surgical intervention groups among patients with strangulated internal hernia-related small bowel obstruction

## Discussion

Internal hernia represents one of the most treacherous etiologies of small bowel obstruction, characterized by an abrupt transition from simple incarceration to strangulated closed-loop ischemia [9, 10]. Our study highlights a critical clinical dilemma: surgical delay in strangulated IH-related SBO is frequently not caused by diagnostic uncertainty regarding radiological severity, but rather by the deceptive absence of classical peritoneal signs. In our cohort, the lack of overt peritonitis emerged as the strongest independent predictor of delayed surgical intervention (OR = 0.021), underscoring the inherent danger of relying on physical examination alone in the early phase of mesenteric compromise.

Our multivariate analysis reveals a critical functional dichotomy between biochemical markers that help address this diagnostic blind spot. While lactate (OR = 3.975) and D-dimer (OR = 3.412) emerged as independent predictors of strangulation, their clinical utility lies primarily in confirming established ischemic injury. In contrast, BE was uniquely identified as a significant factor associated with surgical delay (OR = 1.274). Previous studies have mainly emphasized lactate as a marker of established intestinal ischemia, because its elevation typically reflects tissue hypoxia and anaerobic metabolism [11–14]. Nevertheless, lactate may remain falsely normal in the early stage of ischemia, thereby limiting its value for early recognition [12–14]. In contrast, BE reflects systemic metabolic acidosis and may detect evolving hypoperfusion before overt lactate elevation [15, 16]. Other reported biomarkers, including D-dimer and inflammatory indices such as C-reactive protein, NLR, and systemic immune-inflammation index, have demonstrated variable predictive performance and insufficient specificity when applied alone [12, 13, 17–19]. Taken together, these findings suggest a potential temporal distinction between metabolic markers. Hyperlactatemia typically reflects established anaerobic metabolism and clinically significant tissue hypoxia or hypoperfusion [11], whereas a decline in BE may occur earlier in the course of ischemia, potentially signaling evolving metabolic stress before overt lactate elevation [15, 16]. However, given that BE is influenced by systemic physiological factors, it should be interpreted as a supportive parameter within an integrated clinical and radiological framework. Abnormal fibrinogen levels may further contribute to this early metabolic profile, potentially reflecting early endothelial activation and localized microthrombotic processes within the mesenteric microcirculation before the development of overt transmural ischemia [20]. Importantly, laboratory parameters alone are not sufficient to guide clinical decision-making, as they are susceptible to confounding by comorbid conditions and the localized nature of closed-loop ischemia. The danger of this “diagnostic lag” is further exacerbated by radiological pitfalls. Our study therefore underscores the necessity of integrating these findings with radiological assessment to overcome such limitations.

**Fig. 6:**
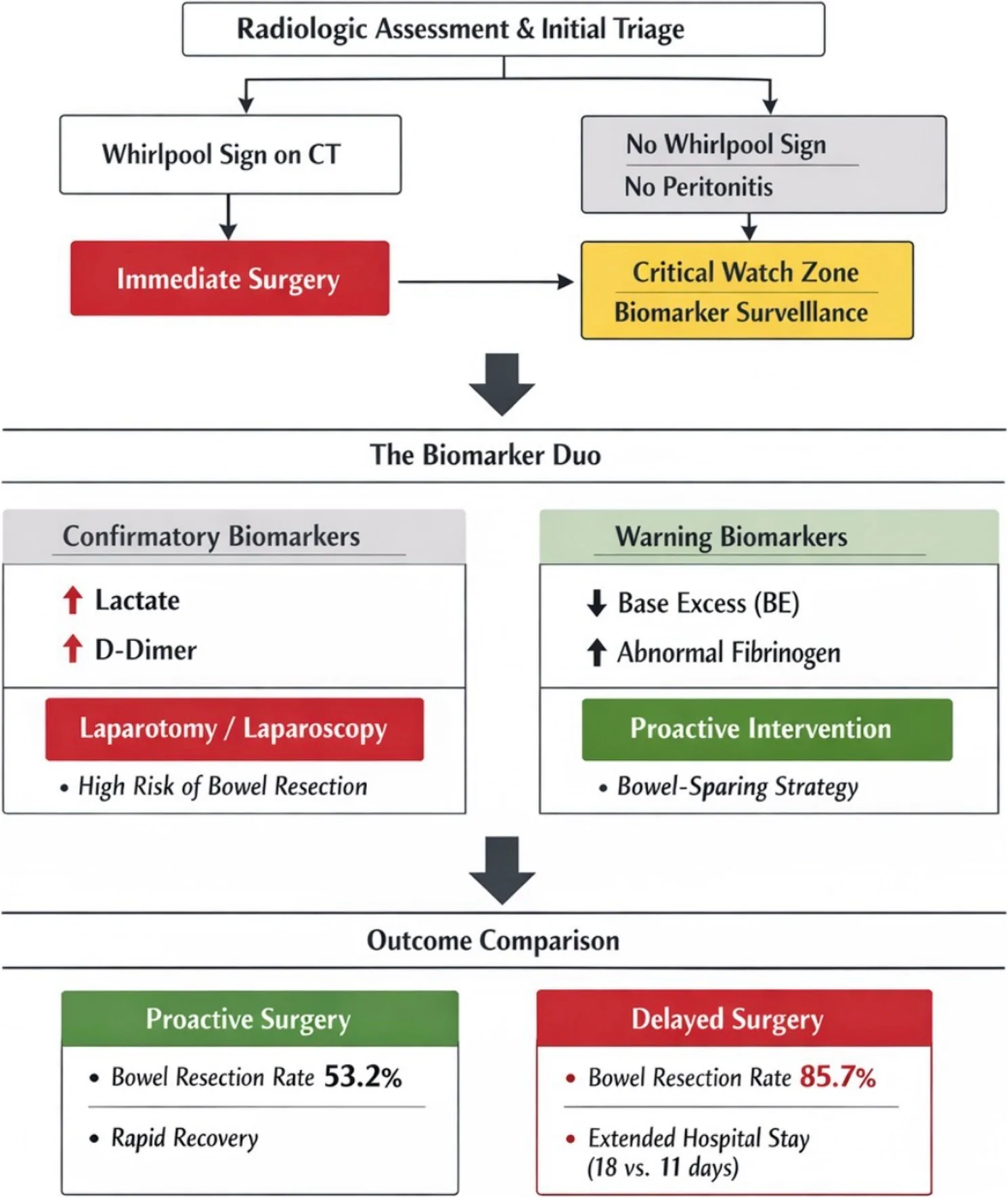
A hypothesis-generating clinical framework integrating radiologic pitfalls and biochemical risk signals in suspected internal hernia. This framework is hypothesis-generating and derived from retrospective analysis and should be interpreted as exploratory rather than a validated clinical algorithm. (1) Initial triage: while the whirlpool sign is typically associated with urgent surgical consideration, its absence (combined with no peritonitis) creates a “Critical Watch Zone” prone to deceptive diagnostic lag. (2) The biomarker duo: to navigate this zone, Warning Biomarkers (declining BE, abnormal Fibrinogen) may signal an early window for proactive intervention, whereas confirmatory biomarkers (Lactate, D-dimer) reflect established injury. (3) Outcome comparison: In our cohort, patients managed without delay demonstrated lower bowel resection rates (53.2% vs. 85.7%) and shorter hospital stays (11 vs. 18 days)

Among imaging features, the whirlpool sign emerged as a significant factor; notably, its absence independently increased the likelihood of delayed intervention (OR = 0.147). This suggests that while the presence of a whirlpool sign prompts heightened urgency, its absence can falsely reassure clinicians, contributing to “watchful waiting” despite ongoing ischemia. In these “silent” cases, where physical and radiological signs are equivocal, abnormalities in BE—potentially together with fibrinogen—may serve as supportive evidence prompting closer clinical attention and earlier consideration of surgery. Collectively, these results support a multimodal diagnostic framework (Fig. 6) in which laboratory markers and imaging findings function synergistically rather than competitively. Practically, this suggests that equivocal clinical findings should not outweigh objective radiological and metabolic warning signals. Integrating CT features with biochemical abnormalities may therefore support earlier operative exploration before strangulation progresses to irreversible bowel injury. Such an approach may help identify high-risk patients before reaching the “point of no return,” potentially supporting earlier operative consideration before progression to irreversible bowel injury. As evidenced by our outcome analysis, this paradigm shift carries profound implications. Given that surgical delay nearly doubled the rate of bowel resection (85.7% vs. 53.2%) and significantly extended recovery, our findings advocate for heightened vigilance toward early metabolic signals. In the management of internal hernia, these findings underscore the potential clinical consequences of delayed intervention, particularly the increased likelihood of bowel resection and prolonged recovery.

### Limitations of the study

This study has several limitations. First, its retrospective, single-center design may introduce selection bias and limit generalizability. In particular, only patients who underwent surgery and had intraoperatively confirmed internal hernia-related small bowel obstruction were included; therefore, non-operatively managed cases and potentially missed cases were not captured. This limits the applicability of the findings to surgically treated patients and may have inflated the observed prevalence of strangulation. In addition, surgical timing was influenced by real-world clinical judgment. Therefore, delay may partially reflect initial clinical assessment of disease severity rather than representing an independent causal effect of time alone. Prospective multicenter validation will be necessary to confirm the predictive value of the proposed framework. Second, laboratory parameters were measured only at admission and may not reflect dynamic metabolic changes; their specificity may also be affected by comorbidities or resuscitation status. Third, imaging findings were based on routine clinical reports rather than blinded review. Subtle signs such as the whirlpool sign and mesenteric crowding are known to demonstrate only moderate interobserver agreement, and variability in interpretation cannot be excluded. This approach reflects real-world emergency practice, in which surgical decisions rely on immediate radiological assessment. However, it may underestimate the optimal diagnostic performance of CT under controlled, blinded review conditions. Fourth, the limited sample size and number of delayed events restricted model complexity and may have reduced statistical precision, resulting in less stable multivariable estimates and wider confidence intervals. External validation was not performed. In addition, some odds ratios, particularly extreme estimates, may have been exaggerated because of sparse data and correlation among markers of clinical severity; therefore, their magnitude should be interpreted cautiously. Finally, long-term outcomes were not assessed. Despite these limitations, the present study provides exploratory evidence supporting the potential value of integrating laboratory and imaging findings in the early assessment of internal hernia-related small bowel obstruction.

## Conclusion

Internal hernia–related small bowel obstruction is a time-critical condition in which surgical delay is often driven by deceptively mild clinical and radiological findings during the early ischemic phase. Our findings show that while lactate and D-dimer indicate established strangulation, a decline in base excess—supported by fibrinogen abnormalities—may signal earlier mesenteric compromise, particularly in the absence of peritonitis or classic CT signs such as the whirlpool sign. An integrated interpretation of laboratory and imaging findings may therefore facilitate earlier intervention, reduce bowel resection, and improve clinical outcomes. 

## Supplementary Information

Supplementary Table 1. Multicollinearity diagnostics for risk factors of intestinal strangulation in patients with internal hernia.

## Data Availability

The datasets used and analyzed during the current study are available from the corresponding author on reasonable request.

## Abbreviations

ALB: Albumin
BE: Base excess
BMI: Body mass index
BUN: Blood urea nitrogen
CI: Confidence interval
CK: Creatine kinase
CKMB: Creatine kinase-MB
CT: Computed tomography
ICU: Intensive care unit
IH: Internal hernia
IQR: Interquartile range
LAC: Lactate
LDH: Lactate dehydrogenase
NLR: Neutrophil-to-lymphocyte ratio
OR: Odds ratio
PLR: Platelet-to-lymphocyte ratio
SBO: Small bowel obstruction
SD: Standard deviation
SSBO: Strangulated small bowel obstruction
STROBE: Strengthening the Reporting of Observational Studies in Epidemiology
WBC: White blood cell count
